# CVID Enteropathy Is Difficult To Treat and Shows a Heterogeneous Histopathology

**DOI:** 10.1007/s10875-025-01920-z

**Published:** 2025-09-30

**Authors:** Noah M. Juliana, Mirjam Severs, Jan Willem Marsden, Joris M. van Montfrans, Pauline M. Ellerbroek, Miangela M. Lacle, Virgil A.S.H. Dalm, Amir Abelmoumen, Helen. L. Leavis

**Affiliations:** 1 Department of Rheumatology, Clinical Immunology & Infectious Diseases, University Medical Center Utrecht, Utrecht University, Utrecht, The Netherlands; 2 Department of Gastroenterology and Hepatology, Radboud University Medical Center Nijmegen, Nijmegen, The Netherlands; 3 Department of Pediatric Immunology and Rheumatology, WKZ, UMCU, Utrecht University, Utrecht, The Netherlands; 4 Department of Pathology, University Medical Center Utrecht, Utrecht University, Utrecht, The Netherlands; 5 Department of Immunology, Erasmus Medical Center, Rotterdam, The Netherlands

**Keywords:** Common Variable Immune Deficiency, Enteropathy, Treatment, Endoscopy, Histopathology, Immunosuppressive Medicine.

## Abstract

**Purpose:**

Enteropathy is a non-infectious complication in Common Variable Immune Deficiency (CVID) associated with increased morbidity and mortality. We characterized this group of CVID enteropathy (CVID-E) patients and investigated the effectiveness of immunosuppressive treatments on its clinical course.

**Method:**

We identified patients with CVID-E in two academic teaching hospitals and obtained informed consents. Using electronic patient health care records, we retrospectively collected clinical information in the national Primary immunodeficiency disorder database until 01-2023.

**Results:**

We included 39 patients with CVID-E. Bronchiectasis (69.2%) and lymphoproliferation (46.1%) were the most frequent co-occurring symptoms. The most common endoscopy findings concerned inflammation (72.2%) and erythema (69.4%); The most prevalent histopathologic findings were IBD-like inflammation (55.6%), indiscriminate chronic inflammation (47.2%) and indiscriminate active inflammation (38.9%). We assessed 88 events of treatment response in the 25 treated patients. Overall treatment response was poor, however there were 31 events of remission observed, ranging from partial to sustained remission. Of these 26 were the result of tumor necrosis factor inhibitors (TNFi) or thiopurines, either as monotherapy or in combination with other immunosuppressive treatment. 10 patients achieved complete remission.

**Conclusion:**

In this study, we describe a cohort of CVID-E patients including related comorbidity, clinical course and response to therapy. CVID-E patients frequently develop other, sometimes severe comorbidities. Our study confirms the alleged heterogeneity regarding endoscopic and histopathological findings, and in one third of patients even multiple distinct abnormalities co-occurred in the same biopsy. We found azathioprine and/or TNFi to be the most effective current treatment.

**Supplementary Information:**

The online version contains supplementary material available at 10.1007/s10875-025-01920-z.

## Introduction

Common variable Immunodeficiency (CVID) is one of the most common primary immunodeficiency, with a prevalence of 1 in 25,000 individuals [1–3]. CVID is characterized by infections due to hypogammaglobulinemia, and impaired antibody production following known exposure or vaccination [4]. Although infections are the most common manifestation of CVID, 27.0–48.0% of patients suffer from non-infectious inflammatory complications, such as granulomatous lymphocytic interstitial lung disease (GLILD), liver disease, (auto)immune cytopenia, inflammatory arthritis, lymphoproliferation, and lymphoma.

Additionally, many CVID patients suffer from gastrointestinal (GI) manifestations, which have a serious impact on the patient’s well-being. Non-infectious gastrointestinal disease, also known as CVID enteropathy (CVID-E), is identified in 9.0–24.0% of patients [5–11]. CVID-E is associated with various abnormalities throughout the gastrointestinal tract, such as coeliac disease-like inflammation, IBD-like inflammation, graft versus host disease-like inflammation (GVHD-like), nodular lymphoid hyperplasia (NLH), atrophic gastritis and lymphocytic colitis [9–14]. Although clinical characteristics of CVID-E, including endoscopic and histological findings, have been published, the response to treatment of enteropathy is largely unknown. Diagnosis is often delayed and when medical therapy is considered, adequate treatment choice is challenging [8–12, 14]. A first step in improving future treatment outcomes for CVID-E patients is gaining insights on historical treatment responses. Therefore, we characterized a cohort of CVID-E patients in care of two Dutch academic teaching hospitals until January 2023. We report their demographics, endoscopic and histopathologic features and its clinical course including response to immunosuppressive therapy.

## Materials and methods

### Ethics statement

Ethical approval for this study for all Dutch participants was received from the Medical Ethical Committee of the Erasmus University Medical Centre Rotterdam, the Netherlands (METC: NL40331.078, METC: 2013-026). Written informed consent was obtained from all patients according to the Declaration of Helsinki [15].

### Study population

We identified patients who were currently under follow-up for CVID in the University Medical Center Utrecht and Erasmus University Medical Center Rotterdam using electronic health records (EHR), until January 2023. These patients were then approached during outpatient clinic visits for inclusion in the national PID database.

For this study, patients were included if they met the following criteria:

1. A diagnosis of CVID according to the IUIS classification [16].
2. Chronic GI symptoms (> 3 weeks) for which no infectious cause was found.

Patients were excluded if they met the following criteria:

1. Chronic GI symptoms possibly caused by laxative misuse.

### Data collection

#### Patient data

All data were collected until January 2023. We obtained demographics (gender, date of birth), date of CVID diagnosis and date of onset of enteropathy symptoms from the EHR. Laboratory data were collected as well, including immunoglobulin levels at time of diagnosis, vaccination response and immunophenotyping (first and last measurement). Lastly, we collected data on CVID related complications and divided these into inflammatory, infectious or malignant complications.

#### Endoscopic data

We collected all available reports on endoscopic (gastrointestinal) procedures, regardless of the indication for endoscopy. We collected data on the following endoscopic findings in the stomach, small intestine or colon: erythema, ulceration, NLH, vascular pattern, stenosis, erosion, inflammation and inflammatory polyps.

Inflammation was further subdivided into segmental, continuous and unknown inflammatory patterns.

#### Histopathology data

All available biopsy reports were collected and analyzed. From each report we documented presence of the following features:

- Abnormalities that occur in the stomach: (*Helicobacter pylori* excluded in all samples); atrophic gastritis, reactive gastropathy; microscopic gastritis and intestinal metaplasia.
- Findings which are found in the small intestine: gastric metaplasia and villous atrophy with or without microscopic enteritis.
- Abnormalities found in the large intestines; microscopic colitis.
- Abnormalities that occur at multiple locations in the GI tract; intraepithelial lymphocytosis (IEL), lymphangiectasis, indiscriminate chronic and active inflammation, GVHD-like inflammation, crypt distortion (mostly in colon), intraepithelial neutrophils (indicative of active inflammation), granuloma and apoptosis (if not associated with GVHD-like inflammation).

We classified several co-occurring features as the following major patterns: coeliac disease-like inflammation, IBD-like inflammation, GVHD-like inflammation, microscopic enteritis/colitis and indiscriminative inflammation. For coeliac disease-like inflammation, duodenal localization of IEL and villous atrophy is not necessarily a precondition, if these features are occurring in the small intestines. Additionally, we classified presence of one or more of the following features as IBD-like inflammation: crypt distortion, intraepithelial neutrophils, granuloma, with or without apoptosis. We classified GVHD-like inflammation by the presence of apoptotic enteritis or colitis. Microscopic colitis was subdivided into lymphocytic colitis, collagenic colitis or “not otherwise specified”. We classified co-occurring features as indiscriminate active or chronic inflammation in case in the biopsy report no features compatible with the aforementioned patterns or other characteristics were reported.

#### Clinical course and response to therapy

Retrospectively, the clinical course of enteropathy related symptoms was recorded for each patient alongside the immunosuppressive medication (IS) used over time. Clinical response was assessed based on reduction of symptoms. We analyzed the effect of medication as an event; that is, if a patient had received three consecutive IS for enteropathy, all three responses were collected. We divided IS into local IS as monotherapy or in combination with systemic IS. Local IS included, budesonide and 5-aminosalicylic acid. Systemic IS included systemic corticosteroids, disease-modifying antirheumatic drugs (DMARDs), biologicals or combination therapy. We defined combination therapy as a combination of systemic corticosteroids and/or systemic DMARDs and/or biologicals. Clinical responses were classified into seven categories: “no change”, “secondary loss of response”, “corticosteroid dependent remission” “partial remission”, “complete remission”, “sustained remission” and “unknown” (see table S1 for detailed definitions).

### Statistical Analysis

All statistical analyses were done in JASP 0.16.4.0. Mean, median, standard deviation (SD) and interquartile range (IQR) were calculated for age and immunophenotyping and frequencies for categorical variables. We used logistic regression to assess the relationship between location of inflammation and inflammatory features, and remission on IS.

## Results

### Study populations

We included 39 patients with CVID related enteropathy (Table 1). 46.1% of the study population was female. The median age of this group was 45.0. The median age at which CVID was diagnosed was 33.0 and the median age at the onset of GI symptoms was 34.0. At time of diagnosis, the median IgG level was 3.3 g/L, the median IgA level 0.1 g/L and IgM level 0.3 g/L. We analyzed the first and last measurements of CD4 + T cells, CD8 + T cells, B cells and NK Cells (Table 1). In 18 of the 39 patients next generation sequencing (NGS) had been performed and revealed abnormalities in eight patients: six were considered as variants of unknown significance and two as relevant pathogenic mutations. These two were heterozygous mutations in TNFRSF13B heterozygosity. 25 patients received immunosuppressive medication for enteropathy and 36 patients received immunoglobulin replacement therapy. The variants of unknown significance are displayed in table S2.

**Table 1:** Features

*Patient characteristics*	*N* (%)
Total	39
Female	18 (46.1%)
Age at CVID diagnosis (median, IQR)	33.0 (20.0)
Current age (Median, IQR)	45.0 (10.5)
Age at onset of GI symptoms (median, IQR)	34.0 (14.0)
NGS performed	18 (46.1%)
- No mutation	2 (5.1%)
- Mutation not of relevance to CVID	8 (20.5%)
- VUS	6 (15.4%)
- Relevant pathogenic mutation	2 (5.1%)
IgG before start suppletion (median, IQR) (g/L)	3.3 (3.4)
IgA before start suppletion (median, IQR) (g/L)	0.1 (0.25)
IgM before start suppletion (median, IQR) (g/L)	0.3 (0.487)
Lowest IgA (median, IQR) (g/L)	0.07 (0.19)
**First measurement immunophenotyping**	
CD4**+** (median, IQR, N) (cells/mm3)	750.0 (548.0) {38}
CD8+ (median, IQR, N) (cells/mm3)	390.0 (202.5) {35}
CD19+ (median, IQR, N) (cells/mm3)	115.0 (230.0) {35}
CD56+ (median, IQR, N) (cells/mm3)	134.0 (133.5) {35}
**Last measurement immunophenotyping**	
CD4**+** (median, IQR, N) (cells/mm3)	452.0 (373.5) {27}
CD8+ (median, IQR, N) (cells/mm3)	314.0 (258.0) {25}
CD19+ (median, IQR, N) (cells/mm3)	47.0 (150.0} {25}
CD56+ (median, IQR, N) (cells/mm3)	90.0 (77.0) {21}
Impaired polysaccharide vaccination responses (%, n abnormal/n tested)	89% (25/28)
**Complications**	
Total number of patients with complications	37 (94.9%)
Inflammatory complications apart from enteropathy	22 (56.4%)
Malignant complications	4 (10.3%)
Infectious complications	35 (89.7%)
**Medication**	
Immunosuppressive medication for enteropathy at any time	25 (62.5%)
Immunoglobulin replacement therapy	36 (92.3%)

*CVID *Common variable immunodeficiency; *Ig *immunoglobulin; *IQR *interquartile range; *NGS *next generation sequencing; *VUS *variant of unknown significance

### CVID associated complications

In total, 37 of 39 of patients had other CVID related complications (Table 2). Inflammatory complications occurred in 22 patients, the most common of which were lymphadenopathy/lymphoproliferation (46.1%), splenomegaly (35.9%) and autoimmunity (33.3%). Autoimmunity was further divided into organ specific autoimmunity (17.9%), systemic autoimmunity (7.7%) and immune cytopenia (12.8%). Malignancies occurred in four patients (10.3%) and 69.2% suffered from bronchiectasis. Co-infections with both norovirus and adenovirus (either simultaneously or alternatingly detectable) were reported in respectively four patients (tested in 38 patients), in one patient only adenovirus was detected.

**Table 2:** Disease complications

Complications	N (%)
*Inflammatory complications*	22 (56.4%)
GLILD	11 (28.2%)
Splenomegaly	14 (35.9%)
Liver disease	6 (15.4%)
Granulomatous disease	4 (10.3%)
Lymphadenopathy/lymphoproliferation	18 (46.1%)
Total autoimmunity	13 (33.3%)
Organ specific autoimmunity	7 (17.9%)
Systemic autoimmunity	3 (7.7%)
Autoimmune cytopenia	5 (12.8%)
*Malignant complications*	4 (10.3%)
Solid tumor	2 (5.1%)
Lymphoma/hematological malignancy	2 (5.1%)
*Infectious complications*	35 (89.7%)
Bronchiectasis	27 (69.2%)
Adenovirus infection	5 (12.8%)
Norovirus infection	4 (10.3%)

*GLILD *granulomatous interstitial lung disease

### Endoscopy findings

From 36 patients endoscopy reports were available. 25 patients underwent gastroscopy, 33 colonoscopy and eight sigmoidoscopy (Table 3). In total, we evaluated 189 endoscopy reports. In four CVID-E patients no abnormalities were ever observed endoscopically. Macroscopic signs of inflammation were found in at least one report from 26 of these 36 patients (72.2%). 37.8% of patients showed inflammation of the stomach, 29.7% inflammation of the smaller intestine and 37.8% of the colon. The inflammation pattern is further described in Table 3. The most prevalent findings were erythema and NLH, present in 25 and 18 patients, respectively.

**Table 3:** Endoscopy findings

Endoscopy findings	N (Total number of endoscopies)			
Total number with available endoscopy reports	36 (189)			
Endoscopy type	Gastroscopy	Colonoscopy	Sigmoidscopy	Total of findings
Total number of patients	25 (87)	32 (91)	8 (11)	36 (189)
Normal	17 (36)	21 (29)	4 (4)	26 (69)
Erythema	17 (30)	16 (25)	4 (4)	25 (59)
Ulceration	4 (11)	7 (16)	1 (1)	11 (28)
Micronodular pattern	0	3 (5)	1 (1)	4 (6)
Nodular lymphoid hyperplasia	6 (12)	16 (26)	0	18 (38)
Reduced vascular Pattern	1 (1)	6 (10)	2 (2)	9 (13)
Stenosis	1 (6)	4 (10)	2 (3)	5 (19)
Erosion	12 (14)	5 (5)	0	14 (19)
Inflammation	15 (32)	15 (33)	4 (5)	26 (70)
*Segment*	12 (23)	8 (17)	2 (2)	19 (42)
*Continuous*	1 (3)	2 (3)	2 (2)	4 (8)
*Pattern unknown*	3 (6)	8 (13)	1 (1)	11 (20)
Inflammatory polyps	1 (1)	3 (3)	1 (1)	3 (5)

### Histopathology findings

Histopathology reports of biopsies were available for all collected endoscopies. The results can be found in Table 4. We evaluated 321 biopsy reports. Inflammatory features were observed in 75.0% of the 36 patients. 32.4% of patients had inflammatory abnormalities in their stomach biopsies, 35.1% in the biopsies of the small intestines and 62.1% in the biopsies of the colon.

**Table 4:** Histopathology findings

**Histopathology findings**	***N*** **(Total number of biopsies)**										
**Total of patients with available biopsy reports**	36										
**Location of biopsy**	Total of findings	Stomach	Duodenum	Jejunum	Ileum	Colon	Ascending colon	Transverse colon	Descending colon	Sigmoid colon	Rectum
**Total number of patients**	36 (321)	24 (55)	29 (51)	6 (6)	24 (42)	23 (47)	15 (25)	10 (22)	15 (25)	16 (25)	16 (23)
**Villous atrophy**	10 (24)	0	6 (13)	3 (3)	6 (8)	0	0	0	0	0	0
**IEL**	13 (59)	3 (5)	8 (16)	3 (3)	3 (6)	5 (8)	4 (4)	2 (4)	3 (4)	2 (5)	2 (4)
**Lymphangiectasis**	3 (4)	0	3 (2)	0	1 (2)	0	0	0	0	0	0
**Indiscriminate chronic inflammation**	17 (55)	8 (13)	6 (7)	0	6 (6)	9 (9)	4 (5)	3 (5)	4 (4)	4 (4)	2 (2)
**Indiscriminate active inflammation**	14 (25)	4 (6)	6 (6)	0	3 (4)	3 (3)	1 (1)	2 (2)	1 (1)	1 (1)	1 (1)
**Atrophic gastritis**	4 (18)	4 (18)	0	0	0	0	0	0	0	0	0
**GvHD like inflammation**	4 (18)	1 (1)	2 (3)	1 (1)	1 (1)	3 (3)	1 (2)	1 (2)	1 (2)	1 (2)	1 (1)
**Microscopic enteritis/colitis**	6 (26)	2 (2)	2 (2)	1 (1)	2 (2)	4 (7)	3 (3)	1 (2)	2 (3)	1 (3)	1 (1)
***Lymphocytic enteritis/colitis***	4 (23)	2 (2)	2 (2)	1 (1)	2 (2)	2 (4)	3 (3)	1 (2)	2 (3)	1 (3)	1 (1)
***Collagenic enteritis/colitis***	1 (2)	0	0	0	0	1 (2)	0	0	0	0	0
***Not otherwise specified***	1 (1)	0	0	0	0	1 (1)	0	0	0	0	0
**IBD like inflammation**	20 (101)	4 (10)	2 (3)	1 (1)	6 (10)	14 (23)	8 (11)	7 (11)	7 (10)	10 (12)	9 (10)
***Crypt distortion/chronicity***	16 (54)	0	1 (2)	0	1 (1)	9 (14)	6 (9)	4 (7)	4 (5)	7 (8)	7 (8)
***Intraepithelial neutrophils/active inflammation***	20 (93)	4 (10)	1 (1)	1(1)	6 (10)	13 (20)	8 (11)	7 (10)	7 (10)	9 (11)	8 (9)
***Granuloma***	0	0	0	0	0	0	0	0	0	0	0
***Apoptosis***	6 (28)	0	1(1)	0	1 (1)	6 (7)	3 (4)	2 (4)	2 (3)	3 (4)	3 (4)
**Gastric metaplasia**	5 (6)	0	5 (6)	0	0	0	0	0	0	0	0
**Intestinal metaplasia**	4 (18)	4 (18)	0	0	0	0	0	0	0	0	0
**Reactive gastropathy**	9 (12)	9 (12)	0	0	0	0	0	0	0	0	0

*IEL* Intraepithelial lymphocytosis

In the stomach, reactive gastropathy (37.5%) and indiscriminate chronic inflammation (33.0%) were the most frequent histopathological features of inflammation.

In the small intestines, villous atrophy (30.3%) and IEL (24.2%) were the most frequent histopathological features of inflammation, which fits with ‘celiac disease-like’.

In the colon, intraepithelial neutrophils (58.1%) and crypt distortion (51.6%) were the most frequent histopathological signs of inflammation which are features of IBD-like inflammation.

There were six patients (16,7%) who had no histopathological abnormalities, two of which also had no endoscopic abnormalities (the other four did show macroscopic signs of inflammation).

**Fig. 1:**
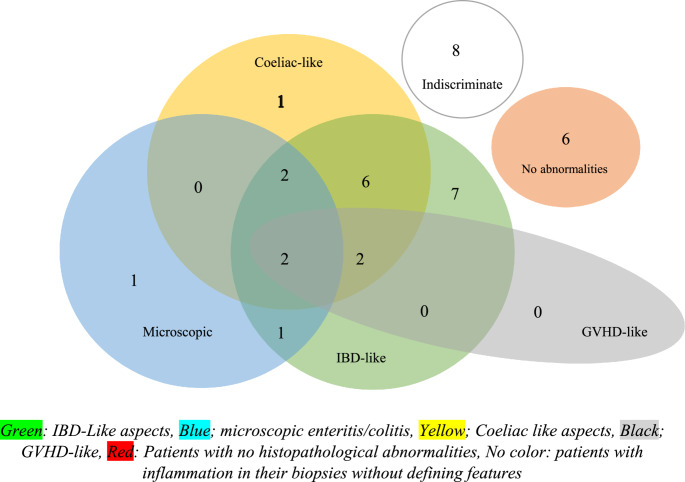
Inflammation patterns and overlap in patients

In 13 patients (36.1%) multiple either concurrent or temporal histopathological patterns occurred at multiple sites of the GI tract. Figure 1 depicts the spread and overlap of histopathologic abnormalities in our cohort.

### Immunosuppressive treatment and response to therapy

In total 25 of 39 patients required IS for enteropathy. During 88 episodes, 17 patients were treated with local corticosteroids, 19 with systemic corticosteroids, 21 with DMARDs, ten with biologicals and ten used combination therapy. DMARDs used in this cohort consisted of the following: methotrexate, 5-aminosalicylic acid, baricitinib, thiopurines (azathioprine and tioguanine), mycophenolate, tacrolimus, thalidomide, cyclosporine, hydroxychloroquine and sulfasalazine. The biologicals consisted of tumor necrosis factor inhibitors (TNFi; adalimumab and infliximab), vedolizumab and ustekinumab. 8.0% of patients remained steroid dependent, and 60.0% of patients did not reach partial, sustained or complete remission despite the use of multiple consecutive IS treatments. The largest number of consecutive IS treatments per patient was nine.

We observed the following responses to IS treatment:

- **local IS as monotherapy**: for 19 episodes in 12 different patients. Five episodes of partial remission were observed on budesonide. Additionally, two complete remissions were recorded in this subgroup on 5-aminosalicylic acid and budesonide respectively.
- **local IS in addition to systemic therapy**: for 14 episodes in 12 different patients. This resulted in one case of partial remission on budesonide and no complete remissions.
- **systemic corticosteroid monotherapy**: for 16 episodes in 13 patients. Partial remission was reached in one episode. Using our definition of complete disease remission when daily corticosteroid use was successfully tapered to below the equivalent of 10 mg prednisone per day, we observed no complete remissions.
- **DMARD monotherapy**: for 15 episodes in 11 different patients. Here we observed one episode of partial remission on methotrexate and two episodes of complete remission on thiopurines. Furthermore, in this subgroup we observed four episodes of sustained remission, all of which were maintained with thiopurines.
- **biological monotherapy**: for eight episodes in six patients. We observed one partial remission, two complete remission and 1 sustained remission, all treated with TNFi.
- **combined therapy**: for 29 episodes in ten patients. This resulted in six episodes of partial remission, these episodes included the following treatments: infliximab, prednisone and azathioprine (*n* = 1), adalimumab and hydroxychloroquine (*n* = 1), prednisone and methotrexate (*n* = 1), prednisone and azathioprine (*n* = 1), prednisone and mycophenolate (*n* = 1) and prednisone, methotrexate and cyclosporine (*n* = 1). In these patients, hydroxychloroquine and cyclosporin were primarily prescribed for other inflammatory manifestations than enteropathy (Autoimmune hemolytic anemia, granulomatous thyroiditis and lung disease, immune thrombocytopenia and organ specific autoimmune disease). Lastly, six complete remissions were observed in this subgroup; azathioprine and prednisone (*n* = 2), tioguanine and prednisone (*n* = 1), prednisone, methotrexate and cyclosporine (*n* = 1), mycophenolate, prednisone and infliximab (*n* = 1) and prednisone and baricitinib (*n* = 1).
- **unknown response**: for 20 episodes in ten patients. Due to lack of documentation, and/or other interventions reducing intestinal symptoms (resection of the intestines, treatment of bacterial co-infection).

For a full oversight of treatment and response see Tables 5 and 6 and S3.

**Table 5:** Immunosuppressive treatment

*Immunosuppressive treatment*	*N*
Total number of patients treated with IS for enteropathy	25
Local corticosteroid	17
Systemic corticosteroid	19
DMARD	21
Biological	10
Combination systemic	10
**Number of treatments for enteropathy per patient**	
0	14
1	4
2	7
3	3
4	2
≥ 5	9
Total number of treatments	88

*IS *immunosuppression; *DMARD *disease-modifying antirheumatic drugs

**Table 6:** IS response

IS response	No change	Secondary loss of response	Corticosteroid dependent remission	Partial remission	Complete remission	Sustained remission	Unknown
Local IS MONO	4	4	0	5	2	0	5
Local IS addition	4	2	0	1	0	0	7
Systemic corticosteroid	4	0	10	1	0	0	1
DMARD	2	2	0	1	2	4	4
Biological	1	3	0	1	2	1	0
Combination	7	3	7	5	6	0	3

*IS *immunosuppression;* Mono *monotherapy

### Predictors of remission

We searched, despite all limitations due to low numbers, for predictors of remission based on disease localization, inflammation type and virus co-infection. Logistic regression analysis failed to establish statistical significance for any of the subanalyses.

Location of inflammation (upper GI versus lower GI).

Ten patients had signs of upper GI inflammation (macroscopic and/or microscopic), for which they received IS. In five (50.0%) of these patients complete remission was achieved. 22 patients had signs of lower GI inflammation for which they received IS. In ten of these patients (45.5%) complete remission was achieved.

In our cohort GI localization does not seem to affect treatment outcome.

Microscopic type of inflammation: Neither number (one to four) nor type of co-occurring inflammation pattern did affect achieving complete remission. Of 20 patients with IBD-like inflammation pattern, 17 were treated with IS, of which eight achieved complete remission (47.0%). Only four patients had a GVHD-like inflammation pattern, and all were treated with IS. Only 1 achieved complete remission (25.0%). 13 patients had Coeliac Disease-like patterns of inflammation. Of these, 12 were treated with IS of which six achieved complete remission (50.0%). Of six patients with a microscopic enteritis/colitis pattern, five received IS and three of them achieved complete remission (60.0%) (Fig. 1).

Norovirus/adenovirus co-infection: in four of five patients with proven norovirus/adenovirus co-infections, no complete remission was achieved. Conversely, only in one of ten patients who achieved complete remission at some point, a virus co-infection was detected. This was the only patient treated with a JAKi.

## Discussion

In this study, we present clinical, endoscopic, histopathologic data and response to therapy in a cohort of 39 CVID-E patients with a very high burden of disease. We found that CVID-E represent a subgroup of immunodeficient patients with a very low serum IgA with a high percentage of bronchiectasis and multiple non-infectious complications. The macroscopic and histopathologic picture is diverse, and abnormalities were found throughout the entire GI tract: multiple concurrent or temporal histopathologic inflammatory patterns could be seen in 36.1% of patients. In our cohort, reaching and maintaining disease remission was challenging, and patients were frequently exposed to a range of consecutive IS medications during the study period. 8% of patients remain steroid dependent, and 60% of patients did not reach complete or sustained remission despite the use of multiple consecutive IS treatments. Steroid-free remission was mostly observed in patients using TNFi (monotherapy as well as combination therapy) or thiopurines.

A significant part of CVID-E patients in our cohort suffered from disease complications (such as bronchiectasis, norovirus and adenovirus co-infections, lymphoproliferation, splenomegaly and auto-immunity). Compared with previous cohorts, our number of disease complications is notably higher [17–19], except for Franzblau et al. who find similar numbers regarding comorbidity [20]. Bronchiectasis occurred frequently in this cohort even when compared to larger CVID cohorts (11.0–37.0%). Despite usually being considered as an infectious complication, due to the high prevalence in this cohort of also inflammatory complications, this may suggest an inflammatory origin to bronchiectasis as well [5, 9, 11, 13]. Alternatively, the cumulative inflammatory complications may require more intensive IS leading to a higher infection burden.

In the vast majority of patients endoscopy revealed macroscopic signs of inflammation (72.2%). This marks that this is a population with proven enteropathy, and not just suffering from functional complaints or infectious conditions. As mentioned earlier, inflammation occurred throughout the GI tract, without a fixed macroscopic inflammatory pattern, as is expected for example in celiac disease or IBD [21–23] When analyzing other studies reporting endoscopy results, we find that endoscopic features vary a lot in CVID-E patients [10, 18].

Consistent with a large variability in macroscopic findings, we also note marked variation in histopathologic findings. In comparison with previous studies, our prevalence of histopathological abnormalities is either similar or higher in our cohort, with the exception of IEL [10, 12, 18]. Only van Schewick et al. reported similar numbers of IEL prevalence, and did not observe granulomas in any of their biopsies [24]. Higher numbers of histopathologic abnormalities may be explained by high disease severity in the cohort.

Inflammatory features were found throughout the entire digestive tract, with either macroscopic inflammation visible during endoscopy or only histopathologic evidence. A spectrum of histopathologic features appears to exist in which some features can be categorized (such as celiac disease-like and IBD-like) but some features also co-occur, although normally considered as separate entities. Features of celiac disease-like, GVHD-like and IBD-like patterns and microscopic enteritis/colitis could be seen within the same patient at different location and times. GVHD-like patterns never co-occurred with IBD-like, and this variation could be attributable to reduced activity of apoptosis over time. Variable severity of granzyme mediated apoptosis probably mediated by cytotoxicity aggravated by norovirus co-infection may also explain this temporal variation in three of our patients [25]. This mixed histopathologic pattern has been recognized as being characteristic of autoimmune enteropathy (AIE) yet anti-enterocyte antibodies against the AIE-75 kDa antigen are lacking in CVID patients and only a subset of patients has the intractable diarrhea characteristic of AIE [26]. In conclusion our patient group shows not only a substantial variety between patients, but individual patients show mixed histopathologic patterns as well [27].

In this cohort two patients had no endoscopic and histopathologic abnormalities, two patients with biopsy findings but no endoscopic findings and four patients with endoscopic findings but no biopsy findings. It could be argued that the patients with minimal to no histopathologic abnormalities did not have enteropathy or that their symptoms were caused by infection or irritable bowel syndrome. However, these patients did receive immunosuppressive therapy for enteropathy. Therefore, without the presence of histopathologic changes, they still may have inflammation as described by Strohmeier et al. [25]. Lastly, there may be sample bias in these cases, or endoscopies were planned after successful treatment.

CVID-E is difficult to manage, which is reflected by the disappointing effect of treatment. In 60.0% of the patients who received IS, no complete remission was reached during the follow-up period, and 8.0% percent stayed steroid- dependent regardless attempts to taper-off steroids. The most often prescribed IS was prednisone, often given in combination with other IS. As monotherapy, however, it performed poorly with only 1 episode resulting in a partial remission. In the rest of the episodes with prednisone monotherapy, prednisone could not be tapered, or active disease remained.

However, treatment with TNFi and thiopurines, either as monotherapy or in combination with other IS, led to the best clinical results in this cohort. About 70% of episodes with azathioprine, monotherapy or combination therapy, resulted in partial, complete or sustained remission; 63.6% episodes of TNFi, monotherapy or combination therapy, resulted in partial, complete or sustained remission as well. Consistent with these results, Franzblau et al. also observed improvement of symptoms on azathioprine and TNFi use, however there was no information available regarding the level of improvement and only 20 of the 67 patients who received treatment, data regarding drug choice and the clinical response was available. TNFi also seemed to have clinical beneficial effect in other case reports and studies [28–31]. Alventosa et al. reported improvement on azathioprine monotherapy in one case, however this resulted in hepatotoxicity. Subsequent switch to TNFi was successful [32]. Vázquez-Morón et al. showed similar results on a combination of azathioprine and adalimumab [33]. The remaining treatment options used in this cohort resulted overall in poor control of disease. More recently, ustekinumab and vedolizumab use has been reported. Ustekinumab therapy resulted in a good clinical response (in 2 of 2 patients), while response to vedolizumab was roughly 50.0% (in 7 of 15 patients) [34–41].

We recently described reaching complete remission induced by treatment with baricitinib in a patient with severe therapy-refractory enteropathy [42]. The use of JAKi may allow a more suitable treatment approach of CVID-E, complementary to the findings of interferon driven inflammation [26, 43].

In this study we analyzed the impact of location of inflammation, inflammation type, and norovirus/adenovirus co-infection on the severity of disease. Although not conclusive, these analyses indicate a trend for worse outcome in patients with GVHD-like inflammation pattern and virus co-infections. Only baricitinib could rescue CVID-E in one patient with GVHD-like inflammation and virus co-infections.

To the best of our knowledge our present study is currently the largest cohort of patients with serious enteropathy necessitating IS, and provides valuable insights into treatment response to different IS classes. In addition, it illustrates the heterogeneity of enteropathy manifested by the range of both endoscopic and histopathologic findings. Based on these findings, future studies of CVID-E should focus on standardizing histopathological classification for enabling comparisons between different disease cohorts and insights into treatment response to optimize treatment outcomes for CVID-E patients [44]. In addition, validation of patient reported outcomes as surrogate for assessing treatment response would largely benefit this patient population.

The largest limitation of our study is its retrospective nature, which, among other factors induces information bias. This could be partially circumvented through future prospective patient cohorts. Hopefully, in future prospective data collection on disease features and treatment response of multicenter, multinational cohorts may provide more reliable evidence for treatment of this heterogeneous patient group in urgent need of effective treatments, whereas RCT would provide the ultimate proof.

In summary, CVID-E patients represent a group of patients with severe complications of disease. This group also showed very low levels of IgA, which coincides with the severe immune dysregulation in this study [45]. Enteropathy is associated with a mixed histopathologic pattern of inflammation, with different histopathological archetypes occurring within the patient group as well as within individual patients. We found thiopurines and TNFi to be the most effective treatment for enteropathy in CVID-E patients. Whether thiopurin induced lymphocytopenia may lead to increased infectious risks, particularly in patients with low T cell counts, and TNFi may be the preferential treatment for CVID-E until JAKi treatment responses are better documented, remains to be proven. Prospective studies and clinical trials are warranted to improve the clinical treatment outcome of this group.

## Supplementary Information

Below is the link to the electronic supplementary material.

ESM 1 (DOCX 23.4 KB)

## Data Availability

No datasets were generated or analysed during the current study.
