# Supplementary material for: CVID Enteropathy Is Difficult To Treat and Shows a Heterogeneous Histopathology
Source: J Clin Immunol. 2025 Sep 30;45(1):129. doi: 10.1007/s10875-025-01920-z (PMC12484083; doi:10.1007/s10875-025-01920-z)
Supplement: Supplementary file 1 — (DOCX 23.4 KB) [file 10875_2025_1920_MOESM1_ESM.docx]

**Supplemental data**

Table S1 Legenda

| **Definitions** |  |
| --- | --- |
| Local IS Mono | Immunosuppressive medication that works locally, such as budesonide and 5-aminosalicylic acid, as monotherapy |
| Local IS addition | Immunosuppressive medication that works locally, such as budesonide and 5-aminosalicylic acid, used in addition to systemic therapies |
| Combination therapy | Combination of 2 or more systemic immunosuppressive medications |
| No change | An episode of immunosuppressive treatment where no change in symptoms are observed |
| Secondary loss of response | An episode of immunosuppressive treatment where after an initial remission of symptoms, the symptoms deteriorate once again. |
| Corticosteroid dependent remission | An episode of remission on immunosuppressive treatment with either systemic corticosteroid as monotherapy or in combination, where dosages of said corticosteroid remains above the equivalent of 10mg prednisone daily. |
| Partial remission | An episode of immunosuppressive treatment which alleviates symptoms caused by enteropathy partially. |
| Complete remission | An episode of immunosuppressive treatment which alleviates symptoms caused by enteropathy completely |
| Sustained remission | An episode of immunosuppressive treatment after an episode which resulted in complete remission, that maintains the remission. |

Table S2 Variants of unknown significance

| Patient | Variants of Unknown Significance |
| --- | --- |
| 1 | NM_138714.3(NFAT5):c.1687T>G, p.(Ser563Ala) |
|  | NM_182972.2(IRF2BP2):c.734-755del22, p.(Ala245fs) |
| 2 | NM_021813.3(BACH2):c.1244G>A, p.(Gly415Glu) |
| 3 | NM_ 006785.4(MALT1):c.2119G>T, p.(Val707Phe) |
|  | NM_001065.4(TNFRSF1A):c.362G>A, p.(Arg121Gln) |
| 4 | NM_006904.7(PRKDC):c.7750T>C, p.(Cys2584Arg) |
| 5 | NM_ 002661.5(PLCG2):c.1938G>T p.(Trp646Cys) |
| 6 | NM_005914.3(MCM4):c.1909C>T, p.(Pro637Ser) |

Table S3 IS therapy episodes and response to treatment for enteropathy in individual CVID patients.

| Patients | Therapy and response | | | | | | | | |
| --- | --- | --- | --- | --- | --- | --- | --- | --- | --- |
| 1 | Aza 4/20-2/21 | Pred 7/21-9/21 | LCS + mesa 9/21-10/21 | TNFi 10/21- |  |  |  |  |  |
|  |  | Mesa 6/21-10/21 |  |  |  |  |  |  |  |
| 2 | Aza + Pred 3/14 – 2/15 | TNFi + aza + pred 2/15-9/15 | TNFi 9/15- 2/17 |  |  |  |  |  |  |
| 3 | Tio 2016 | 3x TNFi 2016 | Vedoluzimab 7/19- 2/20 | Vedoluzimab 10/20-8/21 |  |  |  |  |  |
|  | LCS + mesa 2016 |  |  | Mesa 10/20-12/20 |  |  |  |  |  |
| 4 | pred 6/15-9/15 | Pred + aza 9/15-11/15 | aza 11/15-1/17 | MMF 1/17-3/21 | MMF+ Pred 3/21-6/21 | Pred + tio 6/21-8/21 | tio 8/21- |  |  |
| 5 | Sulfa + Mesa ? | Mesa + Sulfa? |  |  |  |  |  |  |  |
| 6 | Pred + aza 2011-? | aza monotherapie |  |  |  |  |  |  |  |
| 7 | HQ 2/07-1/10 | HQ + pred 1/10-6/14 | HQ +sulfa 2014 | HQ 1/15-5/21 | TNFi + HQ 5/21-8/21 | Pred 08/21-12/21 | Pred + thal 12/21- 6/22 | Pred 6/22- | |
|  |  |  |  | LCS 14-21 |  |  |  |  |  |
| 8 | Pred 10/21-11/21 | LCS 11/21-12/21 | Pred 01/22 | mesa 01/22-06/22 | TNFi 3-2022 |  |  |  |  |
|  |  |  |  | LCS 02/22-5/22 |  |  |  |  |  |
| 9 | Pred + ciclo 14-09/18 | Pred + ciclo + MTX 09/18-5/20 | pred + ciclo 5/20-7/22 | High dosage of pred + ciclo 12/2021 | Pred + ciclo + mtx 7/22-9/22 |  |  |  |  |
|  | LCS 4/18-9/18 |  |  |  |  |  |  |  |  |
| 10 | Pred 11/10 | LCS 11/10-11/11 | Aza 04/11-10/11 | LCS 6/12 | TNFi 6/2012 |  |  |  |  |
| 11 | Mesa 15-18 | LCS 5/2015-9/2015 | pred 02/27-10/27 | TNFi 10/17-10/18 | LCS 9/2018-10/2018 |  |  |  |  |
| 12 | Pred + Tacro 4/08-10/11 | Pred 12/16-02/17 | Pred + MTX 2/17-1/18 | MTX 1/2018-9/2018 ` | Pred + MMF 01/20-01/21 | TNFi + MMF 11/20-3/21 | Pred 3/21-9/21 | Pred + bart 9/21- |  |
|  |  |  | LCS 10/2017-9/2021 |  |  |  |  |  |  |
| 13 | Mesa 93-99 | Pred 97-99 | Aza 11/98-12/14 |  |  |  |  |  |  |
|  |  |  | LCS 98 - 07 |  |  |  |  |  |  |
| 14 | Pred 9/15 | Aza 9/15 |  |  |  |  |  |  |  |
|  | LCS |  |  |  |  |  |  |  |  |
| 15 | Mesa 98 en 00-01 | LCS 03/19-06/19 |  |  |  |  |  |  |  |
| 16 | LCS 11/15-2/16 | Mesa 06/19 |  |  |  |  |  |  |  |
| 17 | Pred 9/15-01/17 |  |  |  |  |  |  |  |  |
| 18 | Pred 08/20-11/20 | TNFi + pred 11/20 |  |  |  |  |  |  |  |
|  | LCS 8/20 |  |  |  |  |  |  |  |  |
| 19 | MMF en pred 3/19-19/19 | MMF + Pred + TNFi 9/19-8/20 | Pred + Tacro 8/20- 9/22 | Pred + tacro + ustekinumab in 5/22 shortlived | Pred + tacro + tio 9/22- |  |  |  |  |
| 20 | Aza + Pred 2014 | Ciclo + pred 9/20-3/21 | MMF + Pred 21-3/22 |  |  |  |  |  |  |
| 21 | Mesa 1999 |  |  |  |  |  |  |  |  |
| 22 | Pred 4/95 |  |  |  |  |  |  |  |  |
|  | Mesa + LCS 4/95 |  |  |  |  |  |  |  |  |
| 23 | Mesa 5/20-7/20 | LCS 7/20-9/20 |  |  |  |  |  |  |  |
| 24 | pred + Sulfa 82-94 | Sulfa 94-98 | Bowl resection, Pred + Sulfa 2001 | Pred + aza 01-04 | Aza 2004- |  |  |  |  |
|  | LCS 82 |  | Mesa 98-05 |  |  |  |  |  |  |
| 25 | Pred 2021 |  |  |  |  |  |  |  |  |

Aza: Azathioprine, Ciclo: Cyclosporine, HQ: Hydroxychloroquine, LCS: Local corticosteroid, Mesa: Mesalazine, MTX: Methotrexate, Pred: Prednisone, Tacro: Tacrolimus, Tio: Tioguanine, TNFi: TNF inhibitor. Red: No change, Purple: Secondary loss of response, Yellow: Corticosteroid dependent remission, Light green: Partial remission, Dark green: Complete remission, Blue: Sustained remission, No color: Unknown response
